# Investigations of Model Multilayer Ceramic Casting Molds in a Raw State by Nondestructive Methods

**DOI:** 10.3390/ma14247761

**Published:** 2021-12-15

**Authors:** Krzysztof Żaba, Sandra Puchlerska, Marzanna Książek, Ryszard Sitek, Paweł Wiśniewski, Jarosław Mizera

**Affiliations:** 1 Department of Metal Working and Physical Metallurgy of Non-Ferrous Metals, Faculty of Non-Ferrous Metals, AGH University of Science and Technology, Al. Mickiewicza 30, 30-059 Krakow, Poland; spuchler@agh.edu.pl; 2 Department of Materials Science and Engineering of Non-Ferrous Metals, Faculty of Non-Ferrous Metals, AGH University of Science and Technology, Al. Mickiewicza 30, 30-059 Krakow, Poland; mksiazek@agh.edu.pl; 3 Faculty of Materials Science and Engineering, Warsaw University of Technology, Woloska 141, 02-507 Warsaw, Poland; ryszard.sitek@pw.edu.pl (R.S.); pawel.wisniewski@pw.edu.pl (P.W.); jmizera@inmat.pw.edu.pl (J.M.)

**Keywords:** multilayer ceramic molds, investment casting, 3D scanning, thermography, CT

## Abstract

This article presents the results of research on the use of modern nondestructive methods such as 3D scanning, thermography and computed tomography (CT) to assess the quality of multilayer ceramic molds. Tests were performed on spherical samples of multilayer ceramic molds in the raw state. Samples were made of molding sands composed of quartz and molochite powders, the alcoholic binder hydrolyzed ethyl silicate (ZKE) and an aqueous binder based on colloidal silica. Thickness measurements of spherical forms were made using a 3D scanner. Porosity measurements were made using CT. Additionally, thermography observations of the mold cooling process were made with controlled temperature and humidity. The results of temperature measurements of samples were compared with measurements of thickness and porosity. The practical goal was to determine the possibility of using thermography, 3D scanning and CT as a quick method for detecting mold defects by varying their thickness, porosity and cracks and for final verification of the ceramic molds’ condition before casting.

## 1. Introduction

In the process of manufacturing aviation parts, in particular critical parts of aircraft engines made of Ni or Co matrix alloys such as low- and high-pressure turbine blades, segments of gas flow guides and turbine directing devices, investment casting technology in multilayer ceramic molds is widely used. This technology enables precise and repeatable production of geometrically complicated shapes of aviation parts. Ceramic molds used in the aviation industry are made of several layers. To produce the ceramic mold, the wax model, which is the subject of separate studies by many authors [1,2,3,4,5,6,7,8], is covered by a ceramic investment compound and ceramic powder alternately. Commonly used materials are aluminosilicate, alumina and silica [9]. In turn, promising materials include yttrium oxide, which can be used for the primary layer and silicon carbide for structural layers [10,11].

The casting mold is expected to have sufficient strength in raw, burned and annealed conditions. It should have adequate heat resistance, creep resistance, size and pore size distribution and thus increased gas permeability. These properties are provided by binders, which are conglomerates in the investment compound. It is expected that binders, combined with the ceramic investment compound and ceramic sands, ensure that the mold will have proper physical and mechanical properties. The basic tests determining the properties of molding sand in the industry are the plate test and viscosity measurement. The plate test determines the amount of adhesive adhering to a plate of specified dimensions. It allows controlling the mass flow, as well as the thickness and quality of the molding mixture covering each layer of the mold. The ceramic slurry cannot be too liquid because it will not cover the wax element evenly, and problems with the proper adhesion of the sprinkle powders will arise. The edges of the models and the recesses are the most sensitive places during the coating. The plate test can also give indirect information about the porosity and the permeability of the first mold layer. It is used to test the consistency of the molding mixture and helps to assess the time needed to properly dry the layer applied to the wax model. The quality of the primary coating of the shell mold determines the quality of the casting surface [12,13]. In addition, the molds should allow easy punching of the casting after the casting process [14].

Mold manufacturing processes are so-called special processes. This means that their results are known after the final product has been inspected. There is a lack of methods and techniques for the effective and efficient quality control of molds, which results in the risk of sending defective molds to the casting process and thus the production of an incompatible product, eliminated only during the final inspection. In the technological process, the quality of molds is assessed visually and by measuring their weight. Comprehensive information on the quality of the mold can only be obtained after it has been broken and after it has been flooded with liquid metal and solidified. Changing the approach to mold quality assessment prior to pouring is very important, as any defects in the mold revealed at this stage of the production process should result in the decision to remove it from the further production cycle. This will reduce production costs due to the smaller number of defective castings. The assessment of the quality of the mold during the process must, for obvious reasons, be carried out by nondestructive methods while ensuring the accuracy and speed of measurements. Among such methods, computed tomography (CT), 3D scanning and thermal imaging may find practical application.

**Figure 1:**
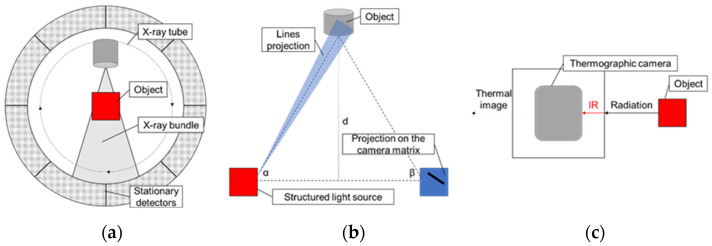
Scheme of the (**a**) CT, (**b**) 3D scanner and (**c**) IR camera.

CT consists in performing flat X-rays of the entire object at a certain interval while rotating the sample for later reconstruction of the image into three-dimensional form. A large part of CT devices operates on the basis of generating cone-shaped radiation. As a result, a two-dimensional radiation layer falls on the sample. The test consists in rotating the object 360° between a stationary lamp and a detector (Figure 1a). The accuracy of the object reconstruction depends on the number of X-rays taken during rotation. Usually, up to several thousand X-rays per revolution are performed, and 3D model reconstruction is performed in the same way as for flat-beam tomography. Because of its ability to detect density, CT is able to separate different material fractions from each other in a complex object [15,16,17,18,19,20]. Until recently, the quality control was limited to tactile and optical measurements, but in the case of the analysis of invisible geometries or material structure, the designers remained helpless. Computed tomography has enabled such measurements to become possible. By performing nondestructive testing on one CT device, one can comprehensively carry out a series of metrological analyses, save time and increase the efficiency and quality of production. An inherent problem of computed tomography is the formation of all kinds of artifacts. Shadows and streaks in X-ray images are a natural effect related to the physical properties of the X-ray beam. Manufacturers are trying to counteract this negative effect by introducing various types of filters that eliminate artifacts even when using a high-power beam.

Castings are characterized by defects in the form of porosity and voids. In turn, cracks are much more common in hard materials such as ceramics. The tomography method had made it is possible to visualize defects, perform geometric measurements or analyze the causes and effects of faults. In the case of the mass production of elements, it is also possible to create software that automatically defines defects in compliance with standards [21,22]. In order to improve the efficiency of product inspection, research is being carried out to improve the accuracy of equipment, the selection of reference objects and others [23,24]. A large amount of research on the porosity of ceramic products is conducted in the context of improving the quality, among others, in terms of porosity visualization and determining the percentage of pores in the volume of the ceramic mold [25]. However, the devices are expensive and usually have small working chambers. The scanning process is extremely thorough, but also very time consuming.

Three-dimensional (3D) scanning is a method that quickly and accurately converts the geometry of three-dimensional objects to digital form (Figure 1b). The resulting digital model can then be edited, processed and used for visualization, prototyping or geometry control by appropriate software. The spatial scanning system is usually a stereoscopic camera system, working together on a triangulation basis, equipped with a light projector, most often LED. The lines projected onto the surface of the tested element are recorded with a minimum of two cameras. A phase shift effect is created based on the sinusoidal intensity distribution on camera matrices. Then, based on the optical transformation equations, 3D coordinates are generated for the pixels of each camera. The measuring accuracy of the system depends on many factors, among others, on the geometrical parameters of the stereoscopic optical system and the size of the measuring field. Full analysis of the performed measurement, including dimensioning of details, comparison with nominal CAD data, color map of dimensional deviations or inspecting sections, is possible with to appropriate software [26]. Software dedicated to 3D scanning provides a wide selection of useful functions, as well as process automation. It also enables direct data exchange with CAD/CAM programs. These methods can be used to control the quality of the semifinished product and the product by comparison with the CAD model, quality control and tool wear and support of assembly processes by virtual control of the alignment degree of individual elements [27,28]. Three-dimensional (3D) scanning has been used in reverse engineering, i.e., the process of obtaining information about the physical product as well as analyzing and processing it in order to develop technical documentation [29]. The advantages of scanners using structured light are very high measurement accuracy of the shape and geometry of objects, high scanning speed and low weight of devices. The limitations of these devices are sensitivity to lighting conditions and problems with scanning outside, and in difficult conditions (sensitivity to shock), it is often required to cover objects with matting spray for glossy surfaces.

Thermography is a research technique based on the detection and analysis of infrared radiation (Figure 1c). The amount of radiation emitted is determined by the temperature of the object. In thermography, electromagnetic waves are usually differentiated by length and frequency. On the thermogram object with a specific temperature, the assigned color is taken to create a map of the temperature distribution on the surface of the examined object. Thermography has become one of the most valuable diagnostic tools in industrial applications. Production and modern construction are important fields of application for thermography because temperature anomalies can indicate potential hazards that can be noticed and repaired early. By controlling individual parts of processes, many qualitative errors can be prevented in a noninvasive way. As a nondestructive technique, thermography allows observation of objects with complex shapes in real time in various configurations [30,31,32]. A relatively new field of thermography application is investment casting for the assessment of ceramic molds drying and cooling processes and for the detection of potential defects in such molds [33]. Thermal imaging cameras have advantages that are limited by generally known and commonly used mercury, resistance or manometric thermometers. IR cameras can be used to determine the temperature in a wide range. Some models measure temperature above 1200 °C. The received signal can be used in recording systems or automation of the technological process; after all, it is a noncontact measurement. IR cameras do not interfere with the measured temperature field. They have low thermal inertia and high accuracy. The main limitation of thermal imaging cameras is the possibility of measuring the temperature of objects only on their surface. The accuracy of the measurements also depends to a large extent on the preparation of the test stand.

The authors of [34,35,36,37,38] undertook preliminary studies on the use of CT, optical 3D scanning and thermography to assess the geometry of wax models and model sets, as well as the structure, porosity and identification of defects in ceramic molds.

The aim of the research presented in publications is to change the general approach to the problem of special processes, in particular the production of multilayer ceramic casting molds. Despite the fact that this process is known and subject to constant research and analysis, as evidenced by recent publications [39,40,41,42,43,44,45,46], it has not been possible to define all the causes of defects, despite detailed monitoring of the manufacturing processes. This issue is extremely important because it concerns the large-scale industrial production of investment castings of aircraft engine parts, which are decisive for flight safety and therefore cannot be burdened with defects.

As a result of using a combination of modern nondestructive tests, i.e., CT, 3D scanning and thermography, it will be possible to transform special processes into measurable ones. The practical aim was to compare the materials used for the production of multilayer ceramic molds in industrial conditions and to determine the possibility of using thermography, 3D scanning and computed tomography as methods of detecting mold defects in the form of various thicknesses, porosities and cracks. The information obtained may be useful for the final verification of the condition of the molds and a possible decision on their removal from the technological process, before casting, which will improve the quality of products and significantly increase economic efficiency.

## 2. Materials and Methods

**Figure 2:**
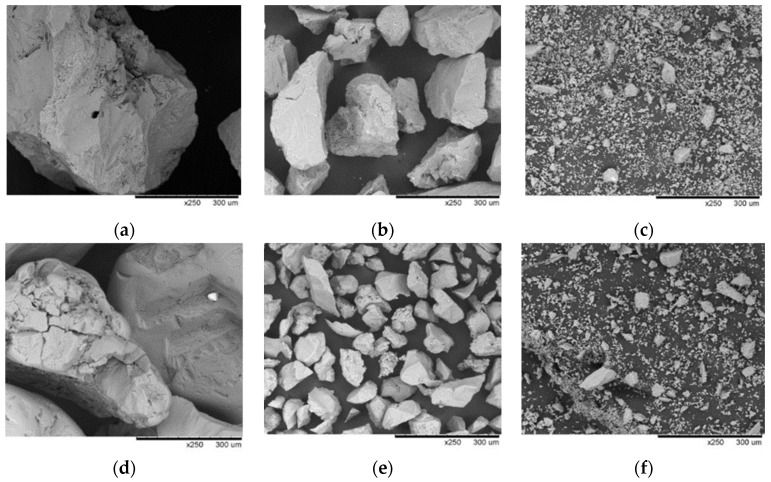
Microstructure of the powder particles used to make the mold samples: molochite sand, 0.5–1.0 mm (**a**); molochite sand, 0.1–0.3 mm (**b**); molochite flour (**c**); quartz sand, 0.5–1.0 mm (**d**); quartz sand, 0.1–0.3 mm (**e**); quartz flour (**f**).

**Figure 3:**
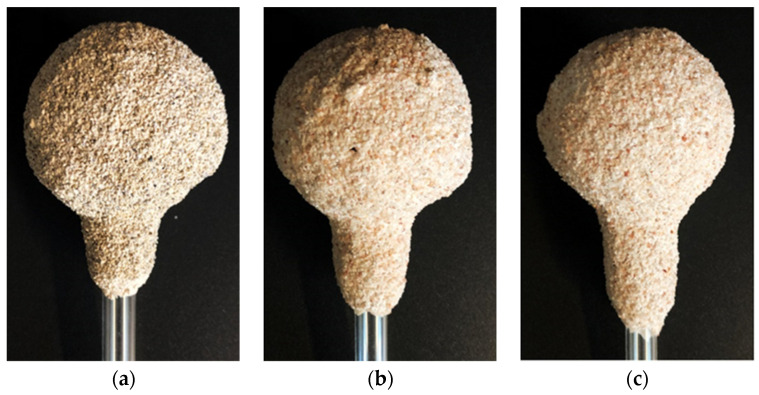
Samples of the ceramic molds: M1 (**a**), M2 (**b**) and M3 (**c**).

The subjects of this study were 3 types of ceramic spherical samples (M1, M2, M3) manufactured by immersion in various mixtures from which casting molds are made in industrial conditions. Molochite flour and quartz flour (Remet, Rochester, UK) were used to obtain the ceramic investment compound. Molochite is an aluminosilicate produced by the calcination of specially selected kaolins mined and refined by Imerys in the Cornwall area. The calcination of molochite at a temperature of 1525 °C not only ensures thermal stability but also gives good creep resistance at normal use temperatures. Quartz, having a melting point of 1715 °C and upper continuous use temperature in the range of 1100–1400 °C, is a very useful material for the fabrication of ceramic mold. Polymer binders and diluents at the same time were hydrolyzed ethyl silicate (ZKE), synthetic amorphous silica (LUDOX PX30) and Remasol Plus and Remasol Premium (Remet, Rochester, UK). Molochite sand and quartz sand with two gradations were used: 0.1–0.3 mm for the first two layers and 0.5–1.0 mm for structural layers. The microstructure of the powder particles used to make the mold samples is shown in Figure 2, while the test samples are shown in Figure 3.

Samples of the ceramic molds M1, M2 and M3, used in the research, are shown in Figure 3.

**Figure 4:**
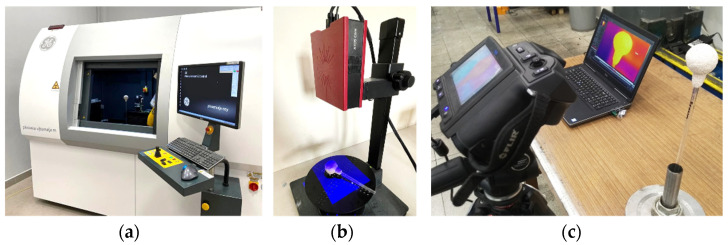
Equipment for NDT testing: computer tomograph Phoenix v|tomex m300 (**a**), GOM ATOS Core 3D scanner (**b**) and Flir T640 thermal imaging camera (**c**).

The CT technique was used to assess the internal discontinuities of the samples produced. The tests were carried out using the high-resolution computer tomography device Phoenix v|tomex m300 (GE dynamic 41|100 detector 410 × 410 mm (16″ × 16″), 100 µm pixel size, 4048 × 4048 pixels (16 MP) for doubled CT resolution) (Figure 4a), enabling the creation of cross-sectional (2D) and spatial (3D) images, which not only allow for a qualitative assessment of the tested material but also allow conducting advanced analyses. The test parameters were: U = 180 kV; I = 220 μA; voxel size, 44.7 μm. The basic result of the tomographic examination, regardless of the device used, is a series of layered X-rays of the examined object. Fiji software was used to analyze the data. The program was used to extract image elements that reflect the porosity of the molds, expose the porosity on tomographic images and calculate the approximate ratio of the porosity to the entire volume of the ceramic mold.

An optical 3D scanning system was used to determine the thickness of the ceramic samples. The tests were performed using a GOM ATOS Core 3D scanner (Figure 4b). The scanner head used for measurements has 2 cameras with a resolution of 5 Mpix and blue LED lighting with a wavelength in the range from 450 to 500 nm, enabling digitization in all light conditions. Scan software was used to control the device, enabling the user to perform the scanning process. The measuring accuracy, verified according to VDI 2634 part 3 and confirmed by an accredited, independent metrology laboratory, was 0.0185 mm. The reference points were glued to the scanning elements. Points enable orientation of the object in space. The samples were scanned, and the obtained measurement data were analyzed in the GOM Inspect software. In order to obtain the thickness of individual ceramic coatings and the wall thickness distribution of the finished mold, the mold scan was compared with the wax model scan by applying them using the referencing function in the GOM Inspect software. The next step was to determine the distance between the mold and model using the appropriate functions. The wall thickness distribution of the multilayer ceramic molds samples was presented as colored deviation maps with values at the marked measurement points.

Thermography was used to determine temperature anomalies during sample cooling. Samples of spherical (M1, M2, M3) and aviation (M2) ceramic molds were examined by passive thermography technique. The value of the emissivity coefficient was 0.95 (typical value for rough ceramic materials). The samples were thermally stimulated by heating in a laboratory dryer at 220 °C for 1 h. The tests were carried out in the laboratory under controlled conditions at a temperature of approx. 21 °C and humidity of 40%. The distance between the camera and the sample was 1 m. After removing the samples from the dryer, the temperature of the ceramic molds was measured using a Flir T640 thermal imaging camera (IR camera) (Figure 4c). The temperature drop during cooling was recorded using the IR camera with a frequency of 7.5 fps. The analysis of measurements was made using the FLIR Research IR software, which allows for import, processing and measurement data analysis. The areas for analysis were marked using ROI, i.e., zones consisting of a certain number of pixels. The IR camera uses image algorithms to build the image and, in effect, presents the temperature assigned to a given pixel. The results are presented in the form of colored temperature maps and graphs.

After completion of testing and analysis according to the above methodology, the comparative analyses using NDT methods were performed.

## 3. Results and Discussion

### 3.1. Results of Chemical Compositions of Molds

The chemical composition of molding coatings is given in Table 1.

### 3.2. Results of CT Research

**Figure 5:**
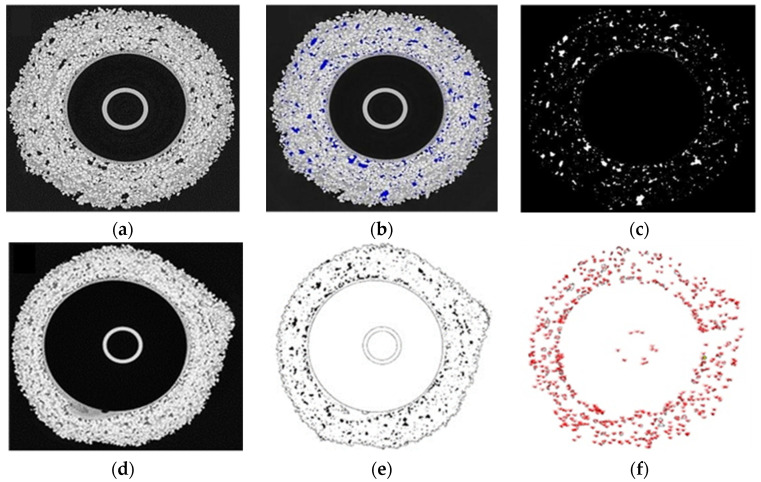
Tomographic image of a spherical sample: immediately after the test (**a**), after exposing the porosity (**b**) and after extracting only the porosity (**c**). Methodology for determining the approximate percentage of the sample’s porosity based on image recognition methods in Fiji: cross-sectional image obtained from the tomograph (**d**), thresholding (**e**) and calculation of porosity (after separation of elements >1 px) (**f**).

With the Fiji software, elements of the cross-sectional images of the molds made using CT were extracted, porosity was exposed (Figure 5a–c) and the approximate ratio of porosity to the total volume of the ceramic mold was calculated. The Fiji software enables such calculations using image recognition methods.

To calculate the percentage of porosity of the spherical samples, the cross-sectional image was binarized, the grayscale of the image was averaged and then the pores, which differ significantly in color from the ceramic mass, were exposed. In the next stage, the number of pixels interpreted as cross-sectional areas of the pores was counted in the program and compared with the number of pixels of the entire image. Such averaged results for all cross-sections made it possible to obtain an approximate percentage porosity of the samples (Figure 5d–f).

**Figure 6:**
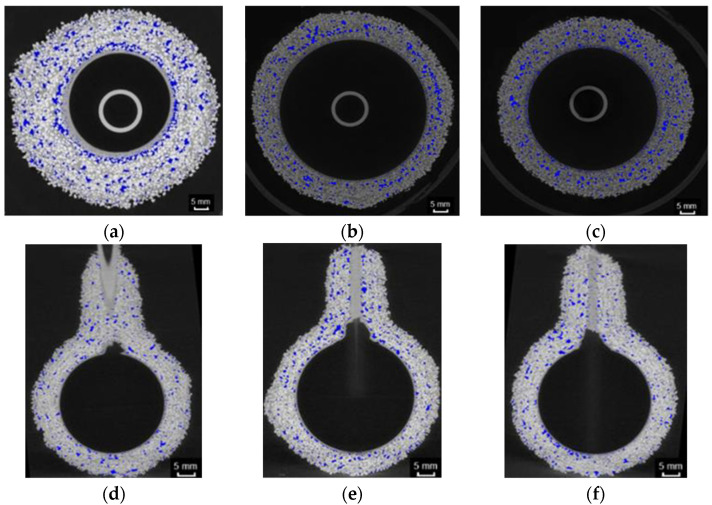
Thickness and porosity of the ceramic samples determined by CT. Cross-section of samples: M1 (**a**), M2 (**b**) and M3 (**c**). Longitudinal section of samples: M1 (**d**), M2 (**e**) and M3 (**f**).

Tomograms obtained for spherical ceramic samples in the raw state (M1, M2, M3) are presented in Figure 6. Due to the distribution of porosity, sample M1 was characterized by the distribution of most pores in the middle layer of the mold. In addition, there was a tendency to have a small number of pores at the base of the spherical sample. Sample M2 had the largest clusters of pores between the model and first structural layer. The M3 sample was characterized by an even distribution of pores. However, larger pores inside the mold wall were noticeable. It is difficult to find a relationship between the fluidity of the materials constituting subsequent structural coatings and the distribution of porosity, as these parameters are the same for samples M1 and M3. All materials had an uneven wall thickness, which is unavoidable in the molding process. Each sphere had a characteristic local thickening of the wall, forming a small cone; however, only in the case of sample M2 were there defects of the modulus layer consisting of local mixing of the first layer material and structural layers.

**Figure 7:**
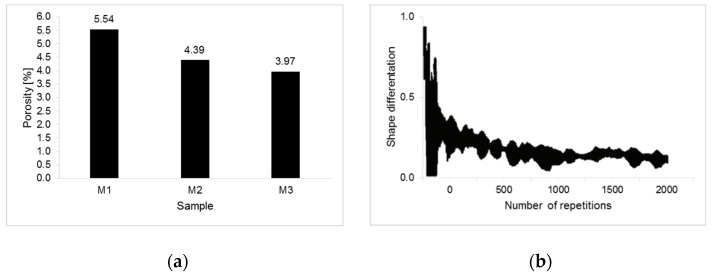
Averaged values of ceramic molds porosity (**a**). Dependence of the porosity shape variation on the number of repetitions (0—similar, 1—different) (**b**).

The properties of the ceramic form are affected by the total volume of the pores and their shape. Using the Fiji software, the porosity was separated from the object and background, which allowed the calculation of the total volume of tomograms individual areas. Figure 7a shows the average porosity results for samples M1, M2 and M3. The calculated averages are 3.97–5.54%, which is the expected range of values for ceramic porous materials prior to heat treatment.

Then, the shape of the pores occurring in individual forms was examined. The results clearly indicate the occurrence of the largest number of pores with similar geometrical features and a spherical-like shape. Figure 7b shows the dependence of geometric differentiation on the number of repetitions (0—similar, 1—different).

**Figure 8:**
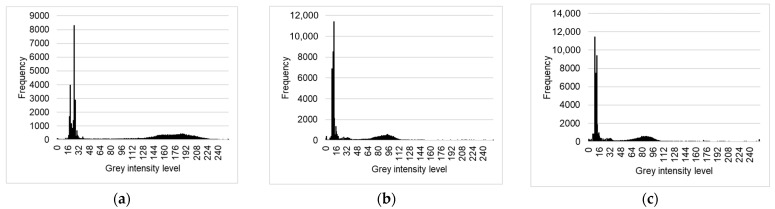
Greyscale histograms for M1 (**a**), M2 (**b**) and M3 (**c**).

Figure 8 shows the count number plots for each grayscale intensity level ranging, determined on the basis of Figure 6, from 0 to 255 for samples M1, M2 and M3.

**Figure 9:**
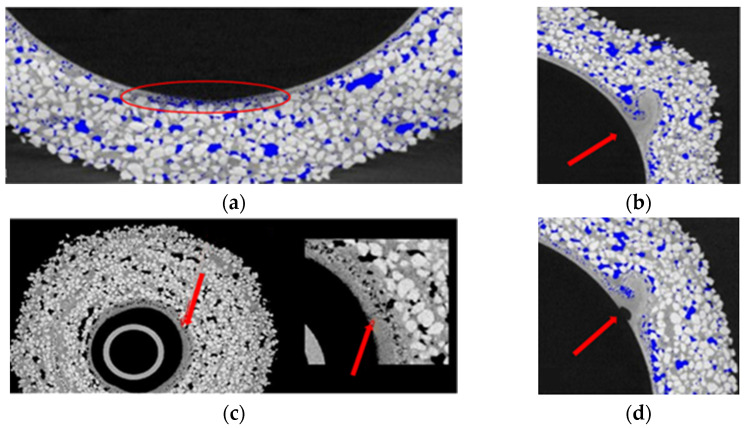

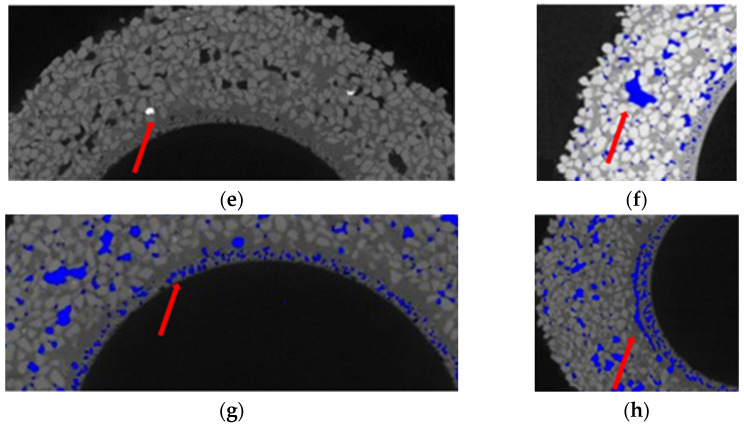
Defined spherical samples defects: open porosity (**a**), irregular material distribution in the first layer (**b**), local fracture of the first layer (**c**), misadjustment of the first layer to the wax model (**d**), material inclusions denser than mass components (**e**), large void (**f**) and local delaminations (**g**,**h**).

In addition to determining porosity, CT allowed the determination of mold thickness (Figure 6) in the range of 7–11 mm and the detection of defects in the samples, including the open porosity (Figure 9a), defects of the first layer (Figure 9b–d), material inclusions denser than mass components (Figure 9e), voids (Figure 9f) and local delamination (Figure 9g,h).

CT tests were used in further tests as a reference base for tests performed with the use of a thermal imaging camera. The results of mold thickness measured with CT were compared in further studies with the results of mold thickness obtained with a 3D scanner.

### 3.3. Results of 3D Scanning Research

**Figure 10:**
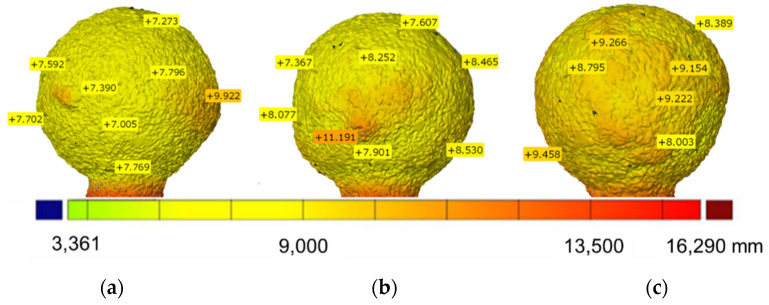
Thickness of the ceramic samples determined by the 3D scanning: M1 (**a**), M2 (**b**) and M3 (**c**).

The comparison of the results of scanning ceramic samples and the wax model allowed obtaining the thickness of the mold. The average thickness of the molds, determined in the GOM Inspect program, depending on the type of material and binder used to make the mold, is, respectively, t_M1_ = 7.342 μm (min. = 7.005 μm, max. 9.922 μm) for M1 (Figure 10a), t_śrM2_ = 8.839 μm (min. = 7.367 μm, max. = 11.191 μm) for M2 (Figure 10b) and t_M3_ = 9.032 μm (min. = 8.389 μm, max. 9.458 μm) The mold thickness test results obtained by the optical 3D scanning method correspond to the thickness results obtained by the CT method (Figure 6).

### 3.4. Results of Thermography Research

**Figure 11:**
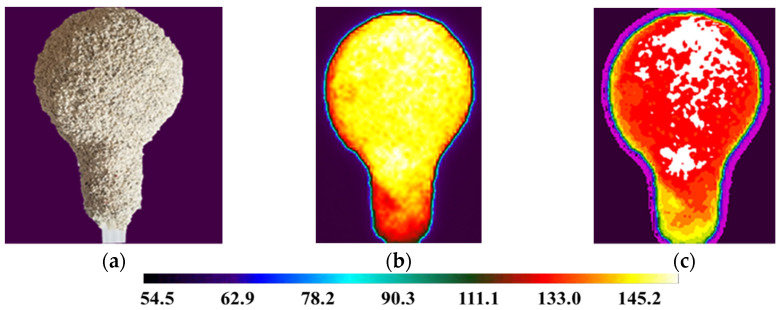
M1 sample (**a**); sample thermogram M1 obtained directly from the camera (**b**); defectogram of M1 sample with a temperature map scale (**c**).

**Figure 12:**
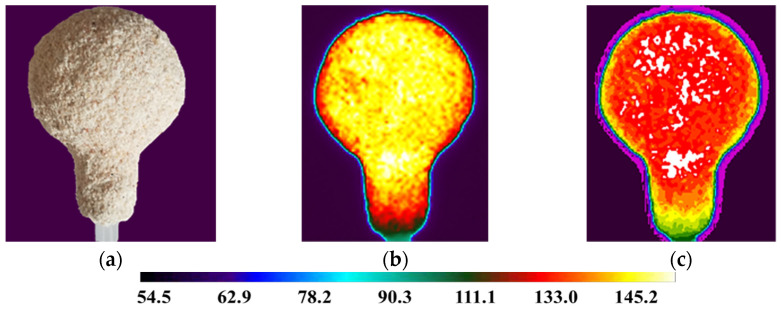
M2 sample (**a**); sample thermogram M2 obtained directly from the camera (**b**); defectogram of M2 sample with a temperature map scale (**c**).

**Figure 13:**
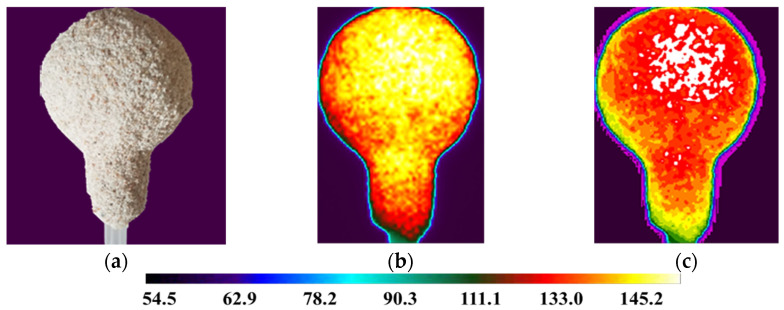
M3 sample (**a**); sample thermogram M3 obtained directly from the camera (**b**); defectogram of M3 sample with a temperature map scale (**c**).

For the analysis using a thermal imaging camera, a fragment of the film showing 5 min of mold cooling was used, starting from the moment of removal from the laboratory dryer. The results of thermography tests are presented in Figure 11, Figure 12 and Figure 13.

**Figure 14:**
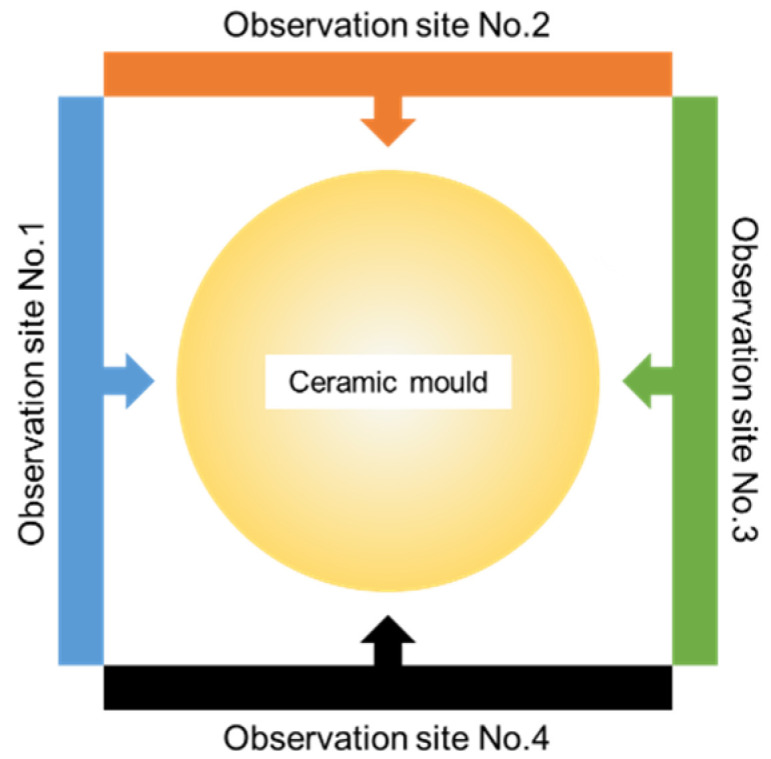
Observation sites of the ceramic mold with IR camera.

Figure 14 shows a diagram of a ceramic mold with the viewing sites using a thermal imaging camera. Temperature measurements were taken on the samples from four sides to make the observation over the entire surface of the samples.

**Figure 15:**
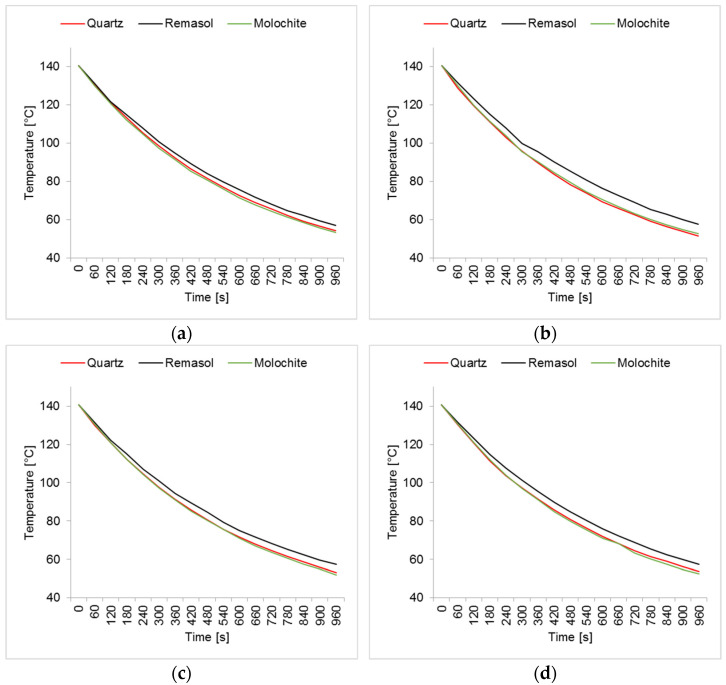
Temperature drop of the ceramic samples depending on the time: (**a**) first site, (**b**) second site, (**c**) third site and (**d**) fourth site.

The results of the temperature drop depending on the material of the form and observation sites are shown in Figure 15.

It can be noticed that samples M1, M2 and M3 had different characteristics of the temperature distribution on their surface. During the 5 min cooling, the average surface temperature of the M1 sample decreased from 189 °C to 121 °C. For sample M2, the temperature dropped from 180 °C to 117 °C. For sample M3, similar values were obtained as for M2 and a decrease from 177 °C to 119 °C. The data are presented on the graph of the dependence of temperature drop on time (Figure 15). Sample M1 had the highest initial and final temperature. For samples M2 and M3, the results were very similar. Sample M1 had a higher temperature in all areas tested but similar temperature drop characteristics. This is the result of a different raw material composition. The solid phase in sample M1 was molochite, while in the other silica, the materials have different thermal conductivity.

### 3.5. Results of Comparative Tests of NDT Methods Used

**Figure 16:**
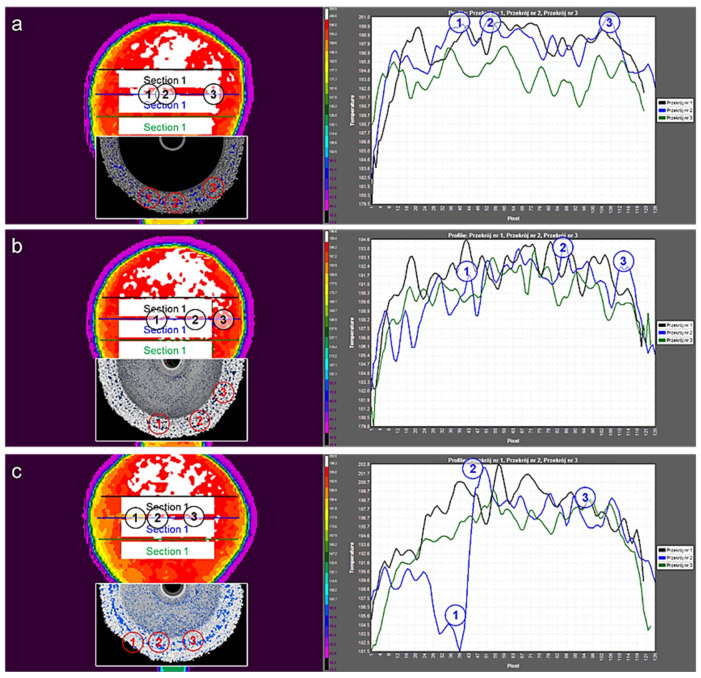
Thermograms and tomograms of spherical samples: M1 (**a**), M2 (**b**) and M3 (**c**).

The defects of M1, M2 and M3 in the form of thickness and porosity deviations from the quality standard are presented on thermograms correlated with tomograms and on the charts of temperature changes depending on the measurement location in three sections in each sample (Figure 16).

An exemplary analysis of recorded thermograms, performed in the ResearchIR program, is shown in Figure 16. In the thermogram of each of the M1-3 samples, three sections were marked to observe temperature changes along each of the straight lines. The digits 1, 2 and 3 denote temperature jumps along the lengths of the sections, both in thermograms and in graphs. Based on the analysis and as a result of comparing the research with other NDT methods, it was found that the temperature jumps indicate a variable thickness of the ceramic mold along the section.

Zones with local fluctuation of the temperature correspond to the smallest thickness of the mold and the highest concentration of porosity. Based on the results of the research, it can be concluded that the tomography method can be used primarily to create quality standards for multilayer ceramic molds for the current range of manufactured products when changing process parameters and when the new process starts. It can also be used to test the quality of the first layer of the mold and discontinuities inside the mold and in parts inaccessible to the 3D scanner, determining the exact percentage porosity and wall thickness of the mold. Tomographic tests can only be used ad hoc due to the inability to perform real-time analysis.

The 3D scanning method can be used to reproduce the thickness of the molds’ ceramic wall to compare the thickness of the mold for two different technologies of applying ceramic coatings and to determine the thickness variation of the mold when changing technological parameters or new process start.

Thermography combines the results of both mold thickness and porosity and is the preferred solution for industrial use for operational process control. After determining the model thickness of the mold by using optical 3D scanning and model porosity for the selected ceramic mixture by using CT, and after correlating the results of the measurement with thermography, it is possible to quantify and qualitatively evaluate the object in real time on the technological line using only thermovision tests.

## 4. Conclusions

Ceramic molds intended for investment casting are made of molding sands. They are characterized by a complex shape, cross-sectional variability, appropriate porosity and mechanical strength. Currently, these types of molds are characterized by classic methods for quality control, and no NDT is used to detect macroscopic defects. This kind of defect can disqualify a defective casting, which affects high production costs and material losses.

Thermal imaging in conjunction with 3D scanning and X-ray tomography analyses can be a valuable test in industrial applications and in combination with other NDT can be part of a system dedicated to effectively detecting defects in a ceramic form before it is flooded with liquid metal. In particular, thermal imaging techniques can be an interesting solution in the modern foundry industry. This technique combines the results of both the thickness of the mold and its porosity and is the preferred solution for use in industrial environments for operational process control. It can be used as a quick technique during the cooling of the mold to the ambient temperature after its firing, i.e., in a duration that allows measuring the batch of molds removed from the furnace.

In the era of growing demand for aircraft engine parts, the ongoing detection of defects in ceramic casting molds will allow for quick, efficient and economic assessment of product quality, which can eliminate material losses of expensive nickel alloys.

## Figures and Tables

**Table 1:** Materials composition of the ceramic samples.

Sample	Coat	Binder	Ceramic Material
M1	1	Ludox PX 30 + molochite flour	molochite sand 0.1–0.3 mm
	2	ZKE + molochite flour	molochite sand 0.1–0.3 mm
	3	Ludox PX 30 + molochite flour	molochite sand 0.5–1.0 mm
	4–7	alternately Ludox PX 30 i ZKE	molochite sand 0.5–1.0 mm
M2	1	hydrolyzed ethyl silicate	quartz sand 0.1–0.3 mm
	2	ZKE + quartz flour	quartz sand 0.1–0.3 mm
	3	Ludox PX 30 + quartz flour	quartz sand 0.5–1.0 mm
	4–7	alternately Ludox PX 30 i ZKE	quartz sand 0.5–1.0 mm
M3	1	Remasol Plus + quartz flour	quartz sand 0.1–0.3 mm
	2	Remasol Plus + quartz flour	quartz sand 0.1–0.3 mm
	3–7	Remasol Premium + quartz flour	quartz sand 0.5–1.0 mm

## Data Availability

The data presented in this study are available on request from the corresponding author.

## References

[1] Bemblage O., Karunakar D.B. A Study on the Blended Wax Patterns in Investment Casting Process. Proceedings of the World Congress on Engineering.

[2] Karwiński A., Młodecki S., Pabiś R., Robak I., Kubosz G. (2011). New generation of pattern materials for investment casting. Arch. Foundry Eng..

[3] Sandhu C.S., Sharma A. (2012). Investigation of Optimize Wax Pattern in the Investment Casting by Using the Different Form of Waxes. IOSR J. Mech. Civ. Eng..

[4] Sharma P., Kasana D., Kumar V., Goel C. (2013). Analysis the Properties of Lost Wax Process and Its Use ability Exploring Possibilities. Int. J. Eng. Sci. Invent..

[5] Guler K.A., Taslicukur Z., Ozer G. (2011). Expanded Polystyrene (EPS) Pattern Application in Investment Casting and Chemical Removing. Ceram. Mater..

[6] Matysiak H., Haratym R., Biernacki R. (2011). Evaluation of wax pattern properties in the lost–wax process. Arch. Foundry Eng..

[7] Zych J., Kolczyk J., Snopkiewicz T. (2012). Methods Investigations of Properties of Wax Mixtures Used in the Investment Casting Technology–New Investigation. Arch. Foundry Eng..

[8] Wawulska-Marek P. (2015). A study on technological properties of investment casting waxes. Adv. Appl. Plasma Sci..

[9] Kolczyk J., Zych J. (2011). High temperature strength of ceramic moulds applied in the investment casting method. Arch. Foundry Eng..

[10] Małek M., Wiśniewski P., Matysiak H., Zielinska M., Kurzydłowski K.J. (2013). Yttrium (III) oxide application for manufacturing prime coat of ceramic shell moulds used in investment casting. Glass Ceram..

[11] Wiśniewski P., Małek M., Matysiak H., Zielinska M., Kurzydłowski K.J. (2014). The Technological Properties of SiC Based Slurries for Manufacturing of Ceramic Shell Moulds for Aerospace Industry. Glass Ceram..

[12] Szeliga D., Kubiak K. (2017). Investigation of casting–ceramic shell mould interface thermal resistance during solidification process of nickel based superalloy. Exp. Therm. Fluid Sci..

[13] Rakoczy Ł., Grudzień M., Cygan R. (2019). Influence of Melt-Pouring Temperature and Composition of Primary Coating of Shell Mould on Tensile Strength and Creep Resistance of Ni-Based Superalloy. J. Mater. Eng. Perform..

[14] Haratym R. (1997). Procesy Odlewania Precyzyjnego w Formy Ceramiczne.

[15] Cantatore A., Müller P. (2011). Introduction to computed tomography. Kgs.Lyngby: DTU Mechanical Engineering.

[16] Cierniak R. (2005). Tomografia Komputerowa: Budowa Urządzeń CT: Algorytmy Rekonstrukcyjne.

[17] Christoph R., Neumann H.J. (2011). X–ray Tomography in Industrial Metrology.

[18] Stock S. (2019). Microcomputed Tomography: Methodology and Applications.

[19] Ratajczyk E., Woźniak A. (2016). Współrzędnościowe Systemy Pomiarowe.

[20] Bauer W., Bessler F.T., Zabler E., Bergmann R.B. (2004). Computer tomography for nondestructive testing in the automotive industry. Dev. X-ray Tomogr. IV Int. Soc. Opt. Photonics.

[21] Ewert U., Fuchs T. (2017). Progress in Digital Industrial Radiology Part II: Computed tomography (CT). Bad. Nieniszcz. Diagn..

[22] Yeung H.Y., Qin L., Lee K.M., Leung K.-S., Cheng J.C.-Y. (2007). Quantification of Porosity, Connectivity and Material Density of Calcium Phosphate Ceramic Implants Using Micro–Computed Tomography. Advanced Bioimaging Technologies in Assessment of the Quality of Bone and Scaffold Materials.

[23] Boden S., Bieberle M., Weickert G., Hampel U. (2008). Three–dimensional analysis of macroporosity distributions in polyolefin particles using X–ray microtomography. Powder Technol..

[24] Schock J., Liebl S., Achterhold K., Pfeiffer F. (2016). Obtaining the spacing factor of microporous concrete using high–resolution Dual Energy X–ray Micro CT. Cem. Concr. Res..

[25] Bartscher M., Neuschaefer-Rube U., Wäldele F. (2004). Computed Tomography—A highly potential tool for industrial quality control and production near measurements. VDI–Berichte.

[26] Mikulski S. (2013). Triangulation method to three-dimensional laser scanners. Comput. Appl. Electr. Eng. Proc..

[27] Helle R.H., Lemu H.G. (2021). A case study on use of 3D scanning for reverse engineering and quality control. Mater. Today Proc..

[28] Yao A.W.L. (2005). Applications of 3D scanning and reverse engineering techniques for quality control of quick response products. Int. J. Adv. Manuf. Technol..

[29] Starnes M.A., Carino N.J., Kausel E.A. (2003). Preliminary Thermography Studies for Quality Control of Concrete Structures Strengthened with Fiber-Reinforced Polymer Composites. J. Mater. Civ. Eng..

[30] Kachel S., Kozakiewicz A., Łącki T., Olejnik A. (2011). Zastosowanie inżynierii odwrotnej do procesu odtwarzania geometrii układu wlotowego silnika RD-33 w samolocie MIG-29. Pr. Inst. Odlew..

[31] Milavanovic B., Pecur I.B. (2016). Review of Active IR Thermography for Detection and Characterization of Defects in Reinfroced Concrete. J. Imaging.

[32] Maldague X.P.V. (1993). Nondestructive Evaluation of Materials by Infrared Thermography.

[33] Koralnik M.K., Wiśniewski P., Moszczyńska D., Mizera J. (2017). Thermovisual estimation of the time necessary for drying the two outer layers of a ceramic casting die. Szkło Ceram..

[34] Wiśniewski P., Sitek R., Koralnik M., Spychalski W., Moszczyńska D., Mizera J. (2017). Investigations of cooling process of ceramic shell samples by using a thermographic camera. Ceram. Mater..

[35] Żaba K., Włodarska J., Puchlerska S. (2018). Selected Methods of NDT Uncertainty Analysis for Quality Rating in Multilayer Ceramic Moulds.

[36] Żaba K., Gracz D., Zych Ł., Książek M., Mizera J. (2018). Porosity Analysis of Multilayer Ceramic Moulds Used in Investment Casting of Aircraft Engine Parts.

[37] Tchórz A., Książek M., Krzak I., Szczepaniak-Lalewicz K., Zaba K. (2018). Evaluation of the internal structure of the multilayer ceramic mould for precision casting critical parts of aircraft engines by X-ray computed tomography. J. Powder Metall. Min..

[38] Żaba K., Balcerzak M., Puchlerska S., Pieja T. (2018). Application of 3D Scanning to Evaluating the Quality of Ceramic Forms.

[39] Liu C., Jin S., Lai X., Wang F., Li F. (2019). Permafrost Analysis Methodology (Pam) For Ceramic Shell Deformation in the Firing Process. Int. J. Metalcast..

[40] Bansode S.N., Phalle V.M., Mantha S.S. (2020). Influence of Slurry Composition on Mould Properties and Shrinkage of Investment Casting. Trans. Indian Inst. Met..

[41] Kanyo J.E., Schafföner S., Uwanyuze S.R., Leary K.S. (2020). An overview of ceramic molds for investment casting of nickel superalloys. J. Eur. Ceram. Soc..

[42] Wiśniewski P., Sitek R., Towarek A., Choinska E., Moszczyńska D., Mizera J. (2020). Molding Binder Influence on the Porosity and Gas Permeability of Ceramic Casting Molds. Materials.

[43] Kumar S., Karunakar D.B. (2020). Characterization and Properties of Ceramic Shells in Investment Casting Process. Int. J. Metalcast..

[44] Kaila V.N., Dave I.B. (2021). The influence of coating sand materials on shell mold properties of Investment casting process. Mater. Today Proc..

[45] Park K., Withey P. (2021). General View of Rhenium-Rich Particles along Defect Grain Boundaries Formed in Nickel-Based Single-Crystal Superalloy Turbine Blades: Formation, Dissolution and Comparison with Other Phases. Crystals.

[46] Pereira J.C., Aranzabe J., Taboada M.C., Ruiz N., Rodriguez P.P. (2021). Analysis of Microstructure and Mechanical Properties in As-Built/As-Cast and Heat-Treated Conditions for IN718 Alloy Obtained by Selective Laser Melting and Investment Casting Processes. Crystals.

